# Grape cultivars adapted to hotter, drier growing regions exhibit greater photosynthesis in hot conditions despite less drought-resistant leaves

**DOI:** 10.1093/aob/mcae032

**Published:** 2024-03-13

**Authors:** Gabriela Sinclair, Erin R Galarneau, Josh F Hnizdor, Andrew J McElrone, Michael Andrew Walker, Megan K Bartlett

**Affiliations:** Department of Viticulture & Enology, University of California, Davis, Davis, CA 95616, USA; USDA-ARS, Plant Genetic Resources Unit (PGRU), Geneva, NY 14456, USA; Department of Viticulture & Enology, University of California, Davis, Davis, CA 95616, USA; Department of Viticulture & Enology, University of California, Davis, Davis, CA 95616, USA; USDA-ARS, Crops Pathology and Genetics Research Unit, Davis, CA 95616, USA; Department of Viticulture & Enology, University of California, Davis, Davis, CA 95616, USA; Department of Viticulture & Enology, University of California, Davis, Davis, CA 95616, USA

**Keywords:** Grapevine, viticulture, osmotic adjustment, osmotic potential, drought tolerance, solute accumulation, inorganic ions, climate change

## Abstract

**Background and Aims:**

Many agricultural areas are expected to face hotter, drier conditions from climate change. Understanding the mechanisms that crops use to mitigate these stresses can guide breeding for more tolerant plant material. We tested relationships between traits, physiological function in hot conditions and historical climate associations to evaluate these mechanisms for winegrapes. We expected a more negative leaf osmotic potential at full hydration (*π*_o_), which reduces leaf turgor loss during drought, and either a metabolically cheaper or more osmoprotectant leaf chemical composition, to allow cultivars associated with hot, dry regions to maintain greater gas exchange in hot growing conditions.

**Methods:**

We measured *π*_o_, gas exchange and leaf chemistry for seven commercially important winegrape cultivars that vary widely in historical climate associations. Vines were grown in common-garden field conditions in a hot wine-growing region (Davis, CA, USA) and measured over the hottest period of the growing season (July–September).

**Key Results:**

The value of *π*_o_ varied significantly between cultivars, and all cultivars significantly reduced *π*_o_ (osmotically adjusted) over the study period, although osmotic adjustment did not vary across cultivars. The value of *π*_o_ was correlated with gas exchange and climate associations, but in the direction opposite to expected. Photosynthesis and *π*_o_ were higher in the cultivars associated with hotter, less humid regions. Leaf chemical composition varied between cultivars but was not related to climate associations.

**Conclusions:**

These findings suggest that maintenance of leaf turgor is not a primary limitation on grapevine adaptation to hot or atmospherically dry growing conditions. Thus, selecting for a more negative *π*_o_ or greater osmotic adjustment is not a promising strategy to develop more climate-resilient grape varieties, contrary to findings for other crops. Future work is needed to identify the mechanisms increasing photosynthesis in the cultivars associated with hot, dry regions.

## INTRODUCTION

Climate change is projected to exacerbate heat and drought stress in many agricultural regions worldwide, with detrimental impacts on crop yield and quality (Lobell *et al.*, 2006; DaMatta *et al.* 2010; Hasegawa *et al.*, 2022). Breeding or genetic engineering of more stress-tolerant cultivars is a promising strategy to mitigate impacts from climate change, but these efforts have been limited by uncertainty around the traits that confer stress tolerance (Vivin *et al.*, 2017; Paleari *et al.*, 2022). Evaluation of trait and climate associations across existing cultivars that are adapted to a diverse range of climatic conditions can identify the traits that have been important for adaptation to hot and dry conditions (Cortés and López-Hernández, 2021).

Two leaf water relationship traits, namely osmotic potential at full hydration (*π*_o_) and osmotic adjustment (Δ*π*_o_), are considered strong predictors of drought performance across cultivars of other crops and wild plant species (Baltzer *et al.*, 2008; Bartlett *et al.*, 2012, 2014, 2016, Blum, 2017) but have not been tested as predictors for stress tolerance in grape cultivars. Both *π*_o_ and Δ*π*_o_ impact drought tolerance by affecting leaf vulnerability to damage from dehydration. Adaptations to reduce damage from dehydration are crucial to maintain gas exchange and carbon assimilation in hot and dry conditions. Much of this damage is caused by the cells losing turgor (i.e. the pressure exerted by water pushing out against the cell walls) as they dehydrate. Turgor supports the cell walls and drives cell expansion (Hsiao *et al.*, 1976; Morgan, 1984). Loss of turgor impairs growth and causes the cell walls to collapse and deform, which impedes water and CO_2_ transport and causes leaves to wilt (Jones and Turner 1980; Scoffoni *et al.*, 2018). The ability to maintain turgor during dehydration is strongly determined by *π*_o_, which is a measure of the potential energy for water influx generated by the cell solutes (Hsiao *et al.*, 1976). Cells with a higher solute concentration exert a stronger driving force for water influx, reducing dehydration and turgor loss. Thus, species or cultivars with higher leaf cell solute concentrations, measured as more negative leaf osmotic potentials at full hydration, typically undergo disruptions in leaf water transport, stomatal closure and wilting under more severe water stress (Baltzer *et al.*, 2008; Bartlett *et al.*, 2016; Scoffoni *et al.*, 2018). Water-stressed plants, including grapevines, can also make leaf osmotic potentials more negative (i.e. osmotically adjust) by accumulating solutes in the leaf cells, which helps to maintain turgor and to reduce leaf vulnerability to wilting, hydraulic dysfunction and stomatal closure (Martorell *et al.*, 2015; Rodriguez-Dominguez *et al.*, 2016; Sorek *et al.*, 2021). Leaf osmotic potentials are typically more negative in plant species adapted to hotter, drier environments, and crop cultivars with greater osmotic adjustment (i.e. larger declines in *π*_o_ under water stress) typically maintain higher yields under drought (Bartlett *et al.*, 2012, Blum, 2017).

Despite the importance of osmotic potential to drought tolerance in other plants, it is largely unknown how osmotic potential and adjustment vary across grape cultivars or impact grapevine performance in dry conditions. Most studies have focused on one or two cultivars and have shown that grapevines adjust osmotically over the growing season or during drought, and that vines that have undergone adjustment are less vulnerable to (i.e. have more negative leaf water potential thresholds for) leaf hydraulic dysfunction and stomatal closure (Martorell *et al.*, 2015; Rodriguez-Dominguez *et al.*, 2016; Sorek *et al.*, 2021). Also, across three cultivars, a more negative *π*_o_ measured once in the growing season was associated with less vulnerability to leaf hydraulic dysfunction and stomatal closure (Dayer *et al.*, 2020). However, the largest study comparing *π*_o_ across cultivars found that osmotic potential was unrelated to stem embolism resistance, raising uncertainty about the importance of this trait to whole-plant drought tolerance (Alsina *et al.*, 2007). Furthermore, other work has found that cultivars typically grown in hotter, drier regions exhibit more water-saving stomatal behaviour, including a lower maximum stomatal conductance (Bartlett and Sinclair, 2021). Modelling work also predicted that osmotic adjustment would increase gas exchange and soil water depletion and cause grapevines to reach critical thresholds for water stress earlier in the growing season (Herrera *et al.*, 2022). These findings suggest that grapevines could use the opposite trait values to wild species (i.e. a less negative osmotic potential and lower osmotic adjustment) to adapt to hotter, drier conditions, if grapevines benefit more from conserving water than maintaining high gas-exchange rates. Evaluation of how these traits contribute to differences in stress tolerance among cultivars would provide insight into whether these traits are worthwhile targets for efforts to improve grapevine cultivars, in addition to the direction in which these traits should be changed.

Previous work has also suggested that the chemical composition of the solutes could impact stress tolerance. Leaf cells can accumulate a wide range of solutes during osmotic adjustment, including inorganic ions, sugars, amino acids and proteins, and solute composition varies widely across species (Zivcak *et al.*, 2016). Synthesizing organic solutes, such as sugars or amino acids, is more resource intensive and energetically expensive than increasing inorganic ion uptake from the soil. Additionally, some organic solutes (e.g. proline) also serve as osmoprotectants, which enhance drought tolerance by stabilizing protein and membrane structures to reduce damage from dehydration (Gagneul *et al.*, 2007; Zivcak *et al.*, 2016). Leaf solute composition has been measured for only a few grape cultivars, and it is unknown whether solute composition contributes to differences among cultivars in drought or heat tolerance (Patakas *et al.*, 2002; Degu *et al.*, 2019). If so, this would indicate that the identification of specific solutes and their role in osmotic adjustment could help to generate new plant material that uses the most effective solutes to achieve optimal values for osmotic potential and osmotic adjustment.

In this study, we tested whether osmotic potential, osmotic adjustment and solute composition vary across *Vitis vinifera* winegrape cultivars historically adapted to different climatic conditions and are associated with differences among cultivars in vine physiological performance (i.e. gas exchange and water potentials) in hot conditions. Specifically, we tested whether: (1) there are significant differences in osmotic potential, osmotic adjustment and solute composition between cultivars; (2) these differences correspond to differences among cultivars in climate associations (i.e. the typical climatic conditions where each cultivar is grown); and (3) these traits are correlated with vine water potentials and gas exchange. We compared these variables across seven cultivars growing in common-garden conditions in a hot wine region. We hypothesized that cultivars that are typically grown in hotter, drier regions would exhibit greater osmotic adjustment and maintain more negative osmotic potentials. We also hypothesized that these traits would enable these cultivars to undergo greater leaf water stress and maintain greater stomatal conductance and photosynthesis over the hottest, most water-stressed period of the growing season. We also expected solute composition to vary across cultivars and correspond to differences in climate associations, although it was unknown from previous work whether adapting to heat and drought stress would favour ion accumulation, as a metabolically ‘cheap’ strategy to lower osmotic potentials, or the production of organic osmoprotectants to protect the biochemical machinery from dehydration. We evaluated relationships between these traits, plant physiological performance and historical climate associations in winegrapes, which are an excellent study system for climate adaptation because cultivars have diverse and well-characterized climatic niches (Anderson and Nelgen, 2020). Furthermore, winegrapes are an economically important crop (valued at $70 billion worldwide) under considerable threat from climate change (Jones *et al.*, 2004; Alston and Sambucci, 2019). Addressing these hypotheses should provide crucial insight into the physiological mechanisms adapting winegrapes to stressful growing conditions.

## MATERIALS AND METHODS

### Plant material and growth conditions

We measured leaf water relationships and chemistry on mature vines of seven *Vitis vinifera* cultivars typically grown in different climatic regions (i.e. Riesling and Pinot Noir from cool regions, Chardonnay, Merlot and Syrah from warm regions, and Zinfandel and Sangiovese from hot regions; *n* = 3 or 4 vines per cultivar). Leaf osmotic potential at full turgor (*π*_o_) was measured on these same vines on three sampling dates throughout the growing season (Table 1).

The vines are established in an experimental vineyard on the University of California, Davis campus (38.53°N, -121.75°W). Half of the vines of each cultivar were divided between two adjacent blocks. The blocks are established with a north–south row orientation and are all trained using a California vertical shoot-positioned trellis system. All vines are grafted onto the same rootstock (420A). Soil types at the site range from a Reiff to a Yolo loam (USGS Web Soil Survey). During the experimental period, all plants received the same irrigation and no precipitation. The vineyard is drip irrigated approximately once per week to replace 80 % of water loss. The replacement amount is based on reference evapotranspiration values generated by the Davis California Irrigation Management Information System (CIMIS) and the seasonal crop coefficient (K_c_) values, which are calculated based on equations from the study by Williams *et al.* (2014). We used the same K_c_ value for all cultivars, because the vines had visually similar canopy sizes, although we did not measure canopy size and thus could have underestimated differences in irrigation demand among cultivars. However, mean predawn water potentials were similar across cultivars (i.e. −0.22 to −0.36 MPa), suggesting that soil water availability was largely consistent across cultivars (Table 2).

**Table 2.** Cultivar mean gas exchange and water potential values over the study period. Gas exchange is measured as stomatal conductance (*g*_s_) and photosynthesis (*A*), and water potentials are measured as predawn (*Ψ*_PD_) and midday water potentials (*Ψ*_MD_). Values are means ± s.e. Letters show Tukey’s post-hoc HSD test results.

Variety	*g*_s_ (mol m^−2^ s^−1^)	*A* (µmol m^−2^ s^−1^)	*Ψ*_PD_ (MPa)	*Ψ*_MD_ (MPa)
Riesling	0.212 ± 0.01^c^	16.27 ± 0.048^b^	−0.31 ± 0.06^a^	−1.3 ± 0.04^b^
Pinot Noir	0.26 ± 0.012^b^	16.26 ± 0.35^b^	−0.22 ± 0.04^a^	−1.06 ± 0.05^a^
Chardonnay	0.25 ± 0.012^bc^	17.06 ± 0.36^ab^	−0.27 ± 0.06^a^	−1.35 ± 0.05^b^
Merlot	0.257 ± 0.011^b^	17.08 ± 0.38^ab^	−0.32 ± 0.05^a^	−1.18 ± 0.06^ab^
Syrah	0.341 ± 0.011^a^	18.36 ± 0.38^a^	−0.36 ± 0.06^a^	−1.17 ± 0.05^ab^
Sangiovese	0.269 ± 0.012^b^	17.37 ± 0.4^ab^	−0.34 ± 0.05^a^	−1.16 ± 0.04^ab^
Zinfandel	0.27 ± 0.011^b^	17.58 ± 0.52^ab^	−0.33 ± 0.05^a^	−1.12 ± 0.05^a^

**Fig. 1.**
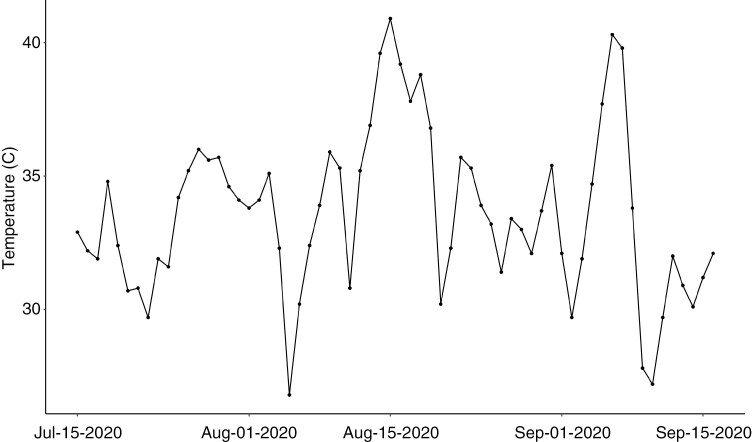
Maximum daily temperatures at the study site over the summer 2020 study period compiled from the University of California, Davis CIMIS station (station #6; https://cimis.water.ca.gov/).

We conducted measurements from the onset of berry ripening (veraison) to harvest (from July to September) in 2020, to capture osmotic adjustment during the hottest period of the growing season. The experimental vineyard is located in a hot (Winkler V) growing region. Daily mean and maximum temperatures ranged from 21 to 31 °C and from 26 to 40 °C over the study period, respectively, based on climate data collected by the Davis CIMIS station (https://cimis.water.ca.gov/). The site experienced a severe heat wave in mid-August (14–18 August 2020) that considerably increased atmospheric evaporative demand (Fig. 1). Following standard commercial practices, we increased irrigation by 50 % in the irrigation event before the heatwave.

### Climate associations

We defined cultivar climate associations in two ways. First, we represented climate as a set of continuous variables, using the methods from Bartlett and Sinclair (2021). To summarize, we used the 2016 global winegrape dataset from Anderson and Nelgen (2020) to identify the Old World growing regions where each cultivar in our study is well represented. For each cultivar, we defined the well-represented regions as those containing ≥5 % of the total Old World bearing area of the cultivar. We then used this subset to calculate the bearing area fraction in each well-represented region, such that the sum of bearing area fractions across well-represented regions equals 100 % for each cultivar. We used the coordinates from Anderson and Nelgen (2020) to extract maximum monthly temperature (*T*_max_) and vapour pressure deficit (VPD_max_) for each growing region from the WorldClim dataset, because these variables were the most strongly correlated with gas exchange in a previous meta-analysis (Bartlett and Sinclair, 2021). We then used the bearing area fractions for each region to calculate a weighted average *T*_max_ and VPD_max_ for each cultivar. We focused on Old World growing regions, where irrigation has historically been banned outright or heavily restricted for winegrapes, to avoid confounding effects of irrigation on the relationships between traits and climate.

Second, to test a common simplified approach, we classified cultivars according to the climate categories from Anderson and Nelgen (2020). This dataset records the global bearing area of each cultivar located in cool, warm or hot growing regions. Mean growing season temperature is <15 °C for cool regions, 17–19 °C for warm regions and >19°C for hot regions. The climate category for each cultivar is defined as the category containing most of its bearing area. Our cultivars were divided among three groups: cool, Riesling and Pinot; warm, Chardonnay, Merlot and Syrah; and hot, Zinfandel and Sangiovese. Similar methods have been used to define regional suitability for cultivars and to predict cultivar responses to future climate conditions (Fraga *et al.*, 2016; Bartlett and Sinclair, 2021; Lamarque *et al.*, 2023). We used both approaches in our study to test whether these methods identify the same relationships between physiology and climate.

### Plant water status and gas exchange

We measured leaf water potential (*Ψ*) and gas exchange at midday (between 1100 and 1300 h) once per week from 16 July to 3 September 2020. We selected healthy, newly expanded mature leaves, 8–12 nodes below the shoot tip, consistently on the east side of the canopy. We measured stomatal conductance and photosynthesis on two leaves per vine with a portable gas-exchange system (Li-Cor 6800; Lincoln, NE, USA), using a fan speed of 10 000 rpm, CO_2_ concentration of 400 µmol mol^−1^ and light intensity of 1900 µmol m^−2^ s^−1^. We allowed humidity and air temperature in the sample chamber to match ambient conditions. We selected two adjacent leaves per vine and measured midday water potential with a pressure chamber (PMS Instrument; model 1505D; *n* = 6–8 leaves per cultivar). Leaves were excised at the base of the petiole, sealed in humidified Whirl-pak bags and either measured immediately or stored in the refrigerator for ≤1 week before measuring. We also measured one leaf per vine for predawn leaf water potential between 0400 and 0600 h at the beginning, middle and end of the experimental period (23 July, 5 August and 3 September 2020).

### Osmotic potential at full turgor

We measured leaf osmotic potential at full turgor (*π*_o_) on three sampling dates (15 July, 18 August and 16 September 2020). We excised one shoot per vine, placed the end of the shoot in deionized water and covered the shoots in a dark, humidified plastic bag to rehydrate overnight. We double-bagged two leaves per shoot in humidified Whirl-pak bags at the same time the following morning to standardize the leaf rehydration time. We then measured leaf osmotic potential following the rapid osmometer method from Bartlett *et al.* (2012). Briefly, we punctured and froze leaf discs in liquid nitrogen, then sealed the discs in a vapour pressure osmometer (Vapro 5600, Wescor, Logan, UT, USA) to determine the osmotic potential at full turgor.

### Sampling for leaf chemistry

To measure leaf solute composition, we collected two leaves per plant from the same shoots used to measure osmotic potential on two of the sampling dates (15 July and 16 September 2020), then flash-froze the leaves in liquid nitrogen. Leaves were cryogenically pulverized to a fine powder using a tissue lyser (Retsch, Newton, PA, USA) with steel jars containing 2-cm-diameter steel balls. Samples were stored at −80 °C until analysis.

### Inorganic ions

K, Ca, Mg and Na ion concentrations were measured by the UC Davis Analytical Lab (Davis, CA, USA), following standard analytical methods (Meyer and Keliher, 1992; Sah and Miller, 1992). Briefly, ions were extracted from 0.4 g of dry leaf biomass using nitric acid–hydrogen peroxide microwave digestion and quantified with inductively coupled plasma atomic emission spectrometry (ICP-AES). Each sample was digested with 2 mL 3X deionized water, 2 mL hydrogen peroxide and 1 mL trace metal grade nitric acid, using a microwave digestion system (Mars Xpress, Matthews, NC, USA). Each sample was brought up to a final volume of 15 mL with 3X deionized water (dilution factor ×30), then diluted ×4 again and analysed with a Thermo ICP 6500 (Thermo Scientific, Waltham, MA, USA). Detection limits for this method range from 0.5 to 100 ppm.

### Amino acids

Amino acids were extracted from 100 mg of fresh leaf tissue using an EZ:FAAST GC-FID kit (Phenomenex, Torrance, CA, USA) following methods from Wallis *et al.*, (2012). Briefly, 100 mg of fresh leaf tissue was extracted in 500 μL of phosphate-buffered saline (PBS) solution adjusted to a pH of 6.8. Samples were vortexed and shaken overnight at 4 °C. The following day, the samples were centrifuged for 1.5 min at 10 000*g*. The supernatant was removed, and the pellet was washed with 500 μL of fresh PBS, centrifuged and left overnight at 4 °C once again. The supernatants were then combined to total 1000 μL. One hundred microlitres of the supernatant collected the following day and was used for amino acid quantification, following the user instructions in the EZ: FAAST gas chromatography–flame ionization detector (GC-FID) kit. The column, eluting medium, reagents and standards used to identify amino acids were all supplied by the kit. Samples were prepared and measured the same day with a Shimmadzu GC-2010 system using an FID.

### Statistical analyses

We used a type III ANOVA to test the model *π*_o_ ~ date + variety + date × variety, to determine whether *π*_o_ varied significantly over the study period (date) and across cultivars (variety) and whether adjustment in *π*_o_ varied significantly across varieties (date × variety). We repeated this analysis for each of the gas-exchange, water potential and solute concentration variables. We were unable to fit a type III ANOVA for stomatal conductance (*g*_s_) and photosynthesis (*A*) because of multicollinearity between the main effects and interaction term; therefore, we tested for main effects of date and variety with a type II ANOVA (Supplementary Data Tables S1 and S2), which has more power for models without interaction terms. For consistency, we also used a type II ANOVA to test the main effects for the other dependent variables with insignificant interaction terms, and this did not impact the significance of the main effects for any of these variables. We used Tukey’s post-hoc HSD tests to compare differences between varieties. We used the same approach to test differences between climate groups (i.e. *π*_o_ ~ date + climate group + date × climate group) (Supplementary Data Tables S3 and S4).

We used linear regression to test correlations between *π*_o_, gas exchange and the predawn (*Ψ*_PD_) and midday water potentials (*Ψ*_MD_). We tested correlations between values measured in the same week, in order to avoid confounding effects from measuring these variables in highly different environmental conditions. We also tested correlations between osmotic adjustment (Δ*π*_o_) and changes in gas exchange and water potential, and between osmotic adjustment at the water potential at the beginning of each adjustment period, to test whether the more water-stressed cultivars exhibited greater adjustment. Finally, we used linear regression to test correlations between the weighted average climate variables and *π*_o_, osmotic adjustment, gas exchange and water potentials. All analyses were conducted with Rstudio (v.4.2.2).

## RESULTS

### Osmotic potential and osmotic adjustment

**Fig. 2.**
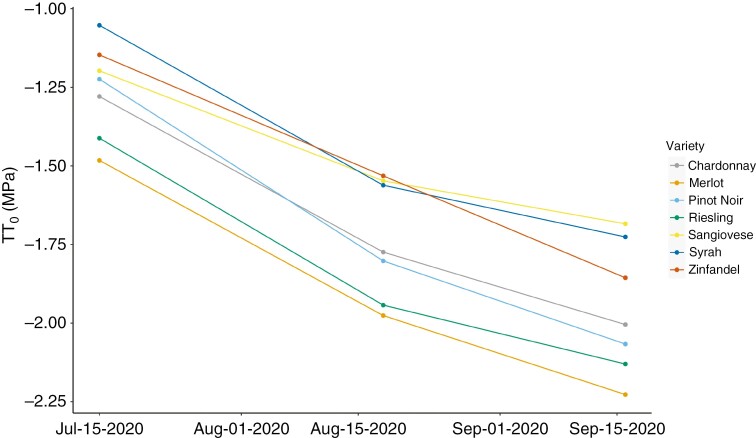
Leaf osmotic potential at full hydration (*π*_o_) measurements from July, August and September. Data points represent the mean *π*_o_ for each cultivar and sampling date (*n* = 6–8). The value of *π*_o_ varied significantly between date, variety and climate group (*P* < 0.05; Tables 1 and 2). However, there was no significant interaction between date and variety or between variety and climate group, indicating that there were no significant differences in osmotic adjustment (Δ*π*_o_) (Tables 1 and 2).

All cultivars significantly reduced osmotic potential at full hydration (*π*_o_) over time, and mean osmotic potential was significantly different across cultivars (ANOVA, *P* < 0.05; Table 1; Fig. 2). However, the interaction between date and variety was not significant, indicating that osmotic adjustment was not different across varieties. Cultivar mean *π*_o_ values ranged from −1.05 ± 0.06 to −1.48 ± 0.08 (mean + s.e.) at veraison (July) and from −1.68 ± 0.05 to −2.23 ± 0.04 at harvest (September). The mean adjustment in π_o_ across cultivars was larger from July to August (Δ*π*_o_ = −0.44 MPa) than from August to September (Δ*π*_o_ = −0.22 MPa) (Table 1). Notably, the ranking in osmotic potential across cultivars was largely consistent over the season (Fig. 2). Mean *π*_o_ was consistently the most negative in Merlot, followed by Riesling, intermediate in Pinot Noir and Chardonnay, and consistently higher in Sangiovese, Syrah and Zinfandel.

**Table 1.** Monthly osmotic potential (*π*_o_) measurements shown in megapascals. Values are cultivar means ± s.e. Letters show Tukey’s post-hoc HSD test results.

Variety	July *π*_o_	August *π*_o_	September *π*_o_
Chardonnay	−1.28 ± 0.09^abc^	−1.77 ± 0.04^b^	−2.0 ± 0.06^bc^
Merlot	−1.48 ± 0.08^c^	−1.98 ± 0.06^b^	−2.23 ± 0.04^c^
Pinot Noir	−1.22 ± 0.06^abc^	−1.8 ± 0.03^b^	−2.07 ± 0.05^c^
Riesling	−1.41 ± 0.04^bc^	−1.94 ± 0.04^b^	−2.13 ± 0.04^c^
Sangiovese	−1.2 ± 0.04^abc^	−1.55 ± 0.05^a^	−1.68 ± 0.05^a^
Syrah	−1.05 ± 0.06^a^	−1.56 ± 0.04^a^	−1.73 ± 0.07^a^
Zinfandel	−1.45 ± 0.09^ab^	−1.53 ± 0.04^a^	−1.86 ± 0.07^ab^

### Plant water status and gas exchange

**Fig. 3.**
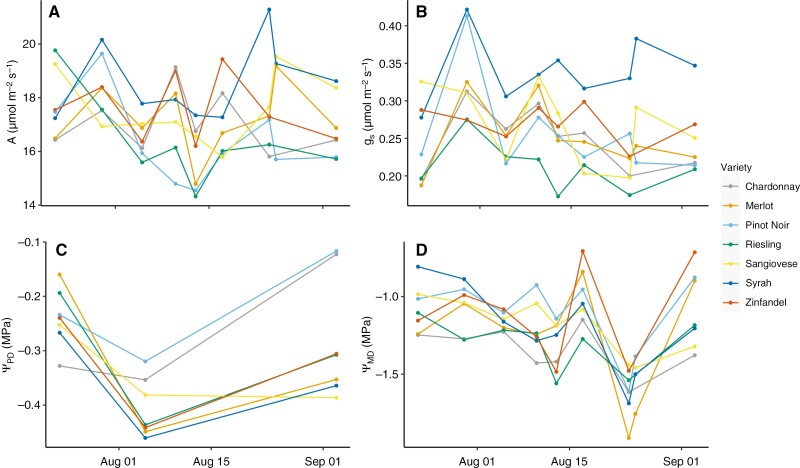
Photosynthesis (*A*; A), stomatal conductance (*g*_s_; B), predawn leaf water potential (*Ψ*_PD_; C) and midday leaf water potential (*Ψ*_MD_; D) measurements over the study period. Points are cultivar means (*n *= 6–8). The values of *A* and *g*_s_ varied significantly between date, variety and climate group, but there was no significant interaction between date and variety or between variety and climate group (Table 2; A, B). Midday water potentials also varied significantly between date and variety but not climate group, whereas there was no significant variation in predawn leaf water potential (Table 2; C, D).

Stomatal conductance (*g*_s_), photosynthesis (*A*) and midday leaf water potentials (*Ψ*_MD_) were significantly different between sampling dates and cultivars (ANOVA, *P* < 0.05; Table 2). Cultivar mean *g*_s_ values from July to September ranged from 0.212 ± 0.010 mmol m^−2^ s^−1^ (mean ± s.e.) for cool-climate Riesling to 0.341 ± 0.011 mmol m^−2^ s^−1^ for warm-climate Syrah. Mean values of *A* ranged from 16.26 ± 0.35 µmol m^−2^ s^−1^ for cool-climate Pinot Noir to 18.36 ± 0.38 µmol m^−2^ s^−1^ for Syrah (Table 2; Fig. 3). Post-hoc tests indicated that *g*_s_ was higher in Syrah than in the other cultivars, while *A* was higher in Syrah than in Riesling and Pinot Noir (Tukey’s HSD, *P* < 0.05; Table 2). Midday leaf water potentials ranged from −1.06 ± 0.05 MPa for Pinot Noir to −1.35 ± 0.05 MPa for Chardonnay and were lower for Chardonnay and Riesling than for Zinfandel and Pinot Noir (Tukey’s HSD, *P* < 0.05) (Table 2; Fig. 3). All cultivars experienced the most negative midday leaf water potentials in late August. In response, there was a wide range in midday leaf water potential from −1.44 MPa (Sangiovese) to −1.83 MPa (Merlot) (Fig. 3). In contrast, predawn leaf water potentials were not significantly different between cultivars or sampling dates.

### Relationships between osmotic potential, gas exchange and midday water potential

**Fig. 4.**
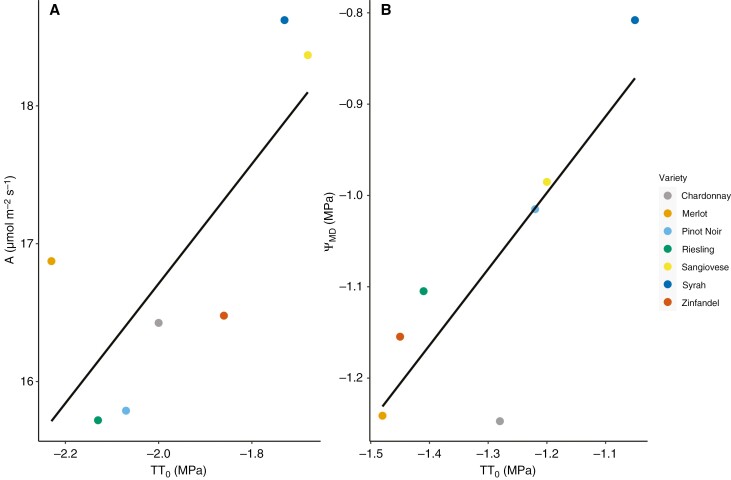
Correlations between osmotic potential (*π*_o_) and significant gas exchange variable (*A*) in the month of September (A) and midday leaf water potential (*Ψ*_MD_) in July (B). (A) The value of *A* was significantly correlated with *π*_o_ across cultivars, but only in the month of September (*r*^2^ = 0.51, *P* < 0.05, *n* = 8). (B) The value of *Ψ*_MD_ was significantly correlated with *π*_o_, but only in the month of July (*r*^2^ = 0.63, *P* < 0.05). Photosynthesis rates were highest in the cultivars with the least negative *π*_o_ values, and *π*_o_ was not significantly correlated with stomatal conductance, contrary to expectation.

We tested correlations between *π*_o_, gas exchange and *Ψ*_MD_ for each of the three sampling periods when these variables were measured in the same week. The value of *π*_o_ was significantly correlated with photosynthesis in September (*r*^2^ = 0.51, *P* < 0.05, *n* = 8; Table 3; Fig. 4A). Stomatal conductance was not significantly correlated with *π*_o_ during the study period. The value of *Ψ*_MD_ was significantly correlated with *π*_o_ early in the season, during the month of July (*r*^2^ = 0.63, *P* < 0.05, *n* = 8; Table 3).

**Table 3.** Linear regressions between osmotic potential and stomatal conductance (*g*_s_), photosynthesis (*A*) and midday water potentials (*Ψ*_MD_) for each sampling date for osmotic potential. Bold values show significant correlations (*P* < 0.05).

Predictor	*P*-value	*r* ^2^
July *g*_s_	0.30	0.05
July *A*	0.99	−0.19
July *Ψ*_MD_	**0.02**	**0.63**
August *g*_s_	0.29	0.07
August *A*	0.36	0.0004
August *Ψ*_MD_	0.57	−0.12
September *g*_s_	0.07	0.41
September *A*	**0.04**	**0.51**
September *Ψ*_MD_	0.48	−0.07

In contrast, osmotic adjustment was not significantly correlated with changes in gas exchange or midday water potential at the beginning of the adjustment period, but the midday water potential at the end of the adjustment period was significantly correlated with osmotic adjustment (*r*^2^ = 0.055, *P* < 0.05, *n* = 6–8; Table 4; Fig. 4B).

**Table 4.** Linear correlations with gas exchange and Δ*π*_o_ and with midday leaf water potential (*Ψ*_md_) and Δ*π*_o_ across all individuals. Bold text signifies significant values.

Linear regression model	*P*-value	*r* ^2^
Δ*g*_s_ ~ Δ*π*_o_	0.8964	−0.012
Δ*A* ~ Δ*π*_o_	0.3912	−0.0031
Δ*Ψ*_md_ ~ Δ*π*_o_	**0.014**	**0.055**

### Leaf chemical composition

**Fig. 5.**
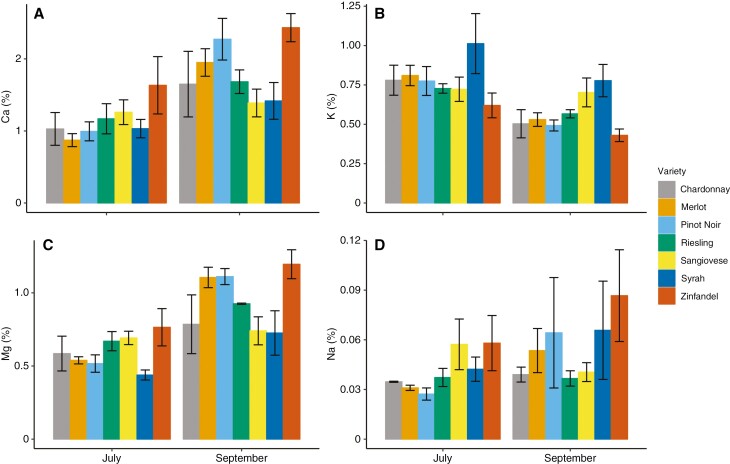
Mean Ca (A), K (B), Mg (C) and Na (D) concentrations, expressed as a percentage of dry leaf sample, at the beginning and end of the sampling period. Bars are means for cultivars and sampling dates (July and September; *n* = 6–8). Error bars represent the s.e. The Ca, K and Mg varied significantly with date, variety and climate group. Only Mg displayed a significant interaction between date and variety. The Na concentration levels were insignificant across all main effects.

All inorganic ion concentrations, except for Na, changed significantly over time, and mean Ca, Mg and K concentrations were significantly different across cultivars (Table 5; Fig. 5). However, the interaction between date and variety, indicating that cultivars showed different patterns in accumulation, was significant only for Mg (Table 5; Fig. 5). Mean Mg and Ca concentrations increased from July to September, whereas K concentrations decreased. The absolute change in concentration was largest for Ca.

**Table 5.** Leaf ion concentrations at the beginning and end of the study period. Values are percentages per dry biomass sample ± s.e. Letters show Tukey’s post-hoc HSD test comparisons.

Variety	Date	Ca (%)	Mg (%)	K (%)	Na (%)
Chardonnay	July	1.03 ± 0.23^a^	0.59 ± 0.012^a^	0.78 ± 0.1^a^	0.03 ± 0.0^a^
Merlot	July	0.87 ± 0.09^a^	0.54 ± 0.02^a^	0.81 ± 0.06^a^	0.03 ± 0.0^a^
Pinot Noir	July	0.99 ± 0.13^a^	0.52 ± 0.06^a^	0.78 ± 0.09^a^	0.03 ± 0.0^a^
Riesling	July	1.17 ± 0.21^a^	0.67 ± 0.07^a^	0.73 ± 0.03^a^	0.04 ± 0.01^a^
Sangiovese	July	1.26 ± 0.17^a^	0.69 ± 0.05^a^	0.72 ± 0.08^a^	0.06 ± 0.02^a^
Syrah	July	1.03 ± 0.13^a^	0.44 ± 0.03^a^	1.01 ± 0.19^a^	0.04 ± 0.01^a^
Zinfandel	July	1.63 ± 0.4^a^	0.76 ± 0.13^a^	0.62 ± 0.08^a^	0.06 ± 0.02^a^
Chardonnay	September	1.65 ± 0.46^a^	0.79 ± 0.2^a^	0.5 ± 0.09^ab^	0.04 ± 0.0^a^
Merlot	September	1.95 ± 0.19^a^	1.11 ± 0.07^a^	0.53 ± 0.04^ab^	0.05 ± 0.01^a^
Pinot Noir	September	2.27 ± 0.29^a^	1.11 ± 0.05^a^	0.49 ± 0.03^ab^	0.06 ± 0.03^a^
Riesling	September	1.68 ± 0.16^a^	0.93 ± 0.0^a^	0.57 ± 0.03^ab^	0.04 ± 0.0^a^
Sangiovese	September	1.39 ± 0.19^a^	0.74 ± 0.1^a^	0.7 ± 0.09^ab^	0.04 ± 0.01^a^
Syrah	September	1.42 ± 0.26^a^	0.73 ± 0.15^a^	0.78 ± 0.1^a^	0.07 ± 0.03^a^
Zinfandel	September	2.43 ± 0.2^a^	1.2 ± 0.1^a^	0.43 ± 0.04^b^	0.09 ± 0.03^a^

**Fig. 6.**
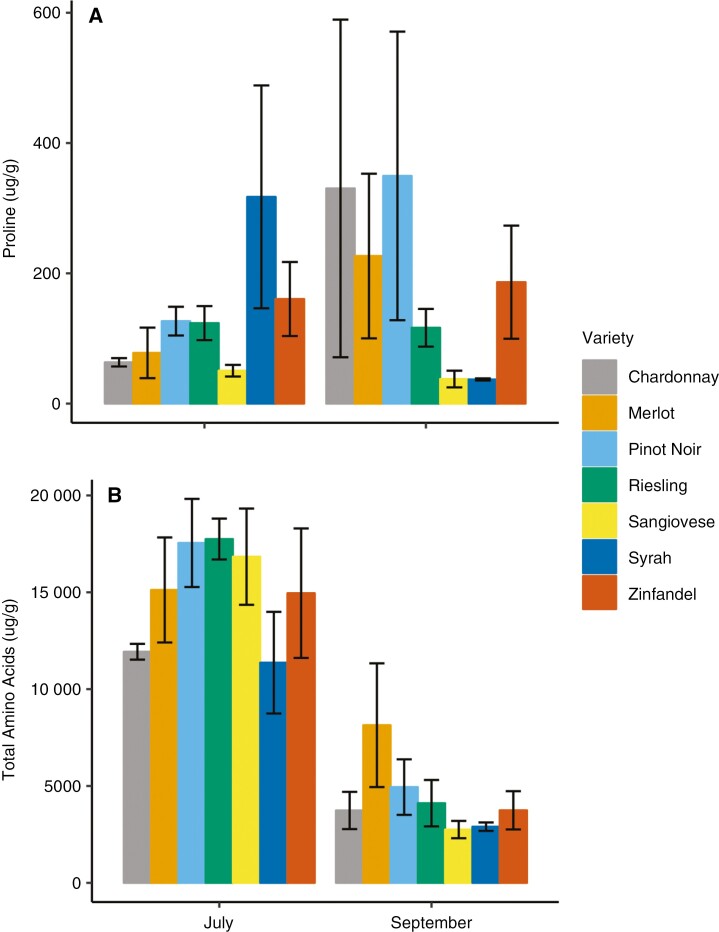
Mean proline (A) and total amino acids (B) concentrations at the beginning and end of the experimental period (July and September). Error bars are the s.e. Proline did not vary significantly between date, variety or climate group, whereas total amino acids varied significantly only with date.

Total amino acid (TAA) content decreased significantly over the season, but mean concentrations were not significantly different across cultivars (Table 6; Fig. 6). Proline concentrations were also not significantly different across cultivars and did not change significantly over time (Table 6; Fig. 6).

**Table 6.** Proline and total amino acid (TAA) concentrations at the beginning and end of the study period.

Variety	Date	Proline (µg g^−1^)	TAA (µg g^−1^)
Chardonnay	July	63.4 ± 6.4^a^	11 931.48 ± 407.18^a^
Merlot	July	77.87 ± 38.78^a^	15 121.09 ± 2711.53^a^
Pinot Noir	July	126.6 ± 22.03^a^	17 547.43 ± 2274.82^a^
Riesling	July	123.58 ± 26.12^a^	17 750.59 ± 1056.66^a^
Sangiovese	July	50.53 ± 8.98^a^	16 839.51 ± 2485.52^a^
Syrah	July	317.38 ± 171.07^a^	11 366.88 ± 2623.6^a^
Zinfandel	July	160.58 ± 56.81^a^	14 953.77 ± 3342.76^a^
Chardonnay	September	330.3 ± 259.11^a^	3737.68 ± 961^a^
Merlot	September	226.58 ± 126.39^a^	8139.59 ± 3195.13^a^
Pinot Noir	September	349.53 ± 221.47^a^	4944.17 ± 1433.79^a^
Riesling	September	116.43 ± 28.93^a^	4113.03 ± 1196.83^a^
Sangiovese	September	37.75 ± 12.79^a^	2751.01 ± 446.7^a^
Syrah	September	37.1 ± 1.65^a^	2900.52 ± 217.4^a^
Zinfandel	September	186.4 ± 86.86^a^	3746.1 ± 988.61^a^

### Climate of origin and climate groups

**Fig 7.**
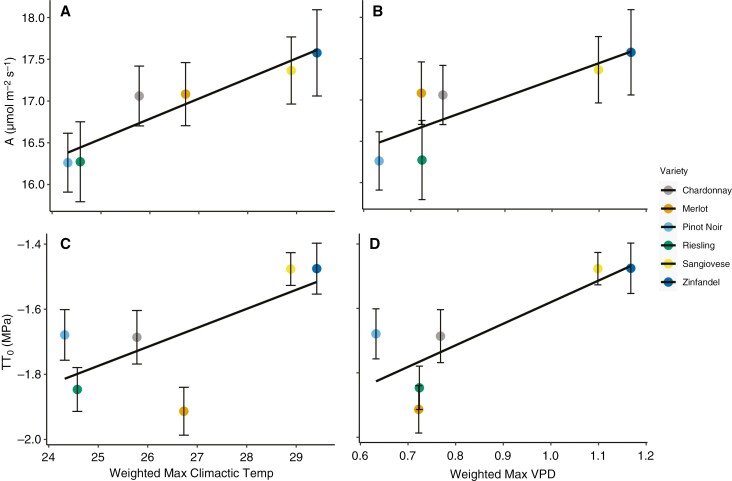
Correlations between *π*_o_ and gas exchange and cultivar climate associations. Climate associations capture growing season climate conditions in the regions where each cultivar is typically grown. Maximum growing season temperature (*T*_max_) and vapour pressure deficit (VPD_max_) were significantly correlated with mean photosynthesis (*r*^2^ = 0.85 and 0.73, respectively, *P* < 0.05, *n *= 8) and *π*_o_ (*r*^2^ = 0.51 and 0.69, *P* < 0.05) over the study period. Cultivars associated with hotter, drier climates had higher photosynthetic rates in our hot common-garden study, but less negative *π*_o_ values, contrary to our predictions.

Photosynthesis and *π*_o_ were significantly correlated with the climate associations of cultivars and were significantly different between categorical climate groups. Photosynthesis was significantly correlated with the weighted maximum growing season temperature (*T*_max_, *r*^2^ = 0.85, *P* ≤ 0.05, *n* = 8) and vapour pressure deficit (VPD_max_, *r*^2^ = 0.73, *P* ≤ 0.05), and *π*_o_ was significantly correlated with VPD_max_ (*r*^2^ = 0.69, *P* ≤ 0.05; Fig. 7). Photosynthesis and *π*_o_ were both higher in the cultivars associated with hot, less humid growing regions. These traits were also significantly higher in the hot-climate cultivars (i.e. Zinfandel and Sangiovese) than the other climate groups (Table 2). Conversely, osmotic adjustment, water potentials and inorganic and organic solute concentrations were not significantly different across the climate groups.

## DISCUSSION

We found that mean osmotic potential varied significantly between winegrape cultivars and that all cultivars reduced osmotic potential (i.e. osmotically adjusted) significantly over the ripening period, but that adjustment was largely uniform, preserving cultivar rankings in osmotic potential (Table 1; Fig. 2). Mean osmotic potentials were correlated with cultivar climate associations, but in the direction opposite to expected, with cultivars typically grown in hotter, less humid wine regions exhibiting less negative osmotic potentials (Table 1; Fig. 7). Depending on the sampling date, osmotic potential and osmotic adjustment were either uncorrelated with gas exchange and leaf water stress or correlated in the direction opposite to expected, with a less negative osmotic potential being associated with greater gas exchange (Tables 1 and 4; Figs 4 and 5). Photosynthesis, but not stomatal conductance, was higher in the cultivars typically grown in hotter, less humid regions (Table 2; Fig. 3). Leaf chemical composition varied between cultivars and over the study period, but this variation was not related to climate associations (Table 5; Fig. 5). Altogether, these findings suggest that reducing leaf osmotic potentials has not been a primary mechanism for winegrapes to adapt to hotter, drier regions, contrary to other plant species (Bartlett *et al.*, 2012). Instead, other mechanisms, such as increasing photosynthetic rates in hot conditions, could be more promising targets for developing climate-resilient grape cultivars.

More negative osmotic potentials increase leaf drought tolerance by improving maintenance of turgor, which reduces leaf vulnerability to wilting, hydraulic dysfunction and stomatal closure during drought (Patakas *et al.*, 1999; Martorell *et al.*, 2015; Scoffoni *et al.*, 2018; Herrera *et al.*, 2022). Thus, we expected cultivars adapted to hotter, drier regions to exhibit more negative mean osmotic potentials and greater osmotic adjustment. However, we found the opposite patterns. Osmotic potentials were significantly less negative for the hot-climate cultivars than for the other climate groups, and less negative osmotic potentials were significantly associated with a higher maximum growing season vapour pressure deficit (VPD_max_) and a higher growing season temperature maximum (*T*_max_) (Fig. 7). These findings could indicate that less drought-resistant leaves are adaptive for winegrapes in hot, dry conditions. We did not find a relationship between *π*_o_ and *g*_s_ in the range of *Ψ* values observed in this study (i.e. mean *Ψ*_MD_ = −1.1 to −1.4 MPa). However, under more severe water stress, a higher *π*_o_ could induce earlier stomatal closure or leaf shedding, producing a larger hydraulic safety margin that extends the time to reach critical thresholds for water stress longer into the growing season (Tyree and Ewers, 1991; Hochberg *et al.*, 2016; Charrier *et al.*, 2018; Herrera *et al.*, 2022). Historically, many European wine regions have had strict legal limitations on irrigation, and larger safety margins could have helped hot-climate cultivars to avoid hydraulic damage that leads to long-term disruptions in function, such as stem embolism, during extreme drought or heat events. Hot-climate cultivars could also require a higher *π*_o_ to produce sufficient safety margins if they are less embolism resistant, which has been found in some studies (Bartlett and Sinclair, 2021) but not others (Lamarque *et al.*, 2023). Alternatively, our findings could indicate that osmotic potential is determined by adaptations beyond drought tolerance. For example, cool-climate cultivars could accumulate more solutes in the leaves during ripening to translocate to the woody tissues before dormancy, to provide greater protection from freezing. Many species use solute accumulation in woody tissues to prevent freezing damage by reducing tissue freezing points and avoiding cellular dehydration (Yuanyuan *et al.*, 2009). Cool-climate cultivars also typically finish ripening and stop translocating sugars and nutrients to the berries earlier in the growing season, which could contribute to greater solute accumulation in the leaves.

All cultivars osmotically adjusted significantly over the ripening period, which is consistent with findings from other field studies for grape (Alsina *et al.*, 2007; Herrera *et al.*, 2022). Most work in other crops has assumed that increasing osmotic adjustment improves drought tolerance (Zivcak *et al.*, 2016; Blum *et al.*, 2017), but we found that osmotic adjustment was not significantly different between climate groups or correlated with climate variables. These findings suggest that osmotic adjustment is not a key trait driving diversification across climates for winegrapes.

Previous work found that cultivars with lower osmotic potentials had more negative water potential thresholds for stomatal closure, and that osmotic adjustment made stomatal and hydraulic conductance less sensitive to leaf water potential over the growing season (Martorell *et al.*, 2015; Sorek *et al.*, 2021; Herrera *et al.*, 2022). Thus, we expected that greater osmotic adjustment and more negative osmotic potentials would allow for greater gas exchange during our study period, in which the vines experienced a record-breaking heatwave at an already hot site. However, *π*_o_ was mostly uncorrelated with gas exchange or correlated in the direction opposite to expected. Osmotic potential was correlated with gas exchange only in July and September, and a less negative osmotic potential was associated with greater *A* and unrelated to *g*_s_ (Table 3). Also, osmotic adjustment was not correlated with changes in gas exchange (Table 4). Altogether, these findings suggest that *π*_o_ and the capacity for maintenance of leaf turgor is not a main driver of cultivar differences in gas exchange in typical vineyard conditions. Instead, osmotic adjustment and *π*_o_ might be more closely related to gas exchange during more severe water stress, closer to thresholds for stomatal closure. Alternatively, previous work has suggested that a higher *π*_o_ allows for a higher maximum *g*_s_ and *A* by reducing maximum turgor in the epidermal cells and, thus, turgor limitations on maximum stomatal opening (Henry *et al.*, 2019). This finding is consistent with the positive correlation between *π*_o_ and *A*, but the lack of a correlation with *g*_s_ suggests that a direct effect of *π*_o_ on stomatal opening is unlikely to drive this relationship. Instead, hot, high VPD conditions might have selected independently for both a higher *π*_o_ and *A*.

Photosynthesis was significantly higher in cultivars typically grown in regions with a higher maximum temperature and VPD (Fig. 7). This is the first study to test correlations between typical growing region climate and *A*, but these findings are largely consistent with previous comparisons of fewer cultivars. Of the six studies where at least two cultivars with published mean growing season temperatures were measured for gas exchange in hot (>30 °C) conditions, both *A* and *g*_s_ were significantly higher for the warmer-climate cultivar in three studies (Moutinho-Pereira *et al.*, 2007; Palliotti *et al.*, 2015; Greer, 2018), only *A* was higher in two (Chaves *et al.*, 1987; Costa *et al.*, 2012), and neither was significantly different in one (Medrano *et al.*, 2003). However, the climate variables were not correlated with stomatal conductance, suggesting that the relationship with *A* was not driven by stomatal behaviour. Instead, the heat-adapted cultivars could have a more heat-tolerant photosynthetic biochemistry. High temperatures (>35 °C) can limit photosynthesis by reducing maximum rates of carboxylation (*V*_cmax_) and the electron transport chain reactions (*J*_max_) (Gallo *et al.*, 2021). The *V*_cmax_, *J*_max_ and their temperature dependence vary between cultivars. For example, *V*_cmax_ and *J*_max_ were more strongly downregulated as temperatures increased above 35 °C in Grenache than in Syrah (Gallo *et al.*, 2021) and in Chardonnay than in Merlot (Greer *et al.*, 2018). The heat-adapted cultivars could have a greater capacity to protect or repair the photosynthetic biochemical machinery from heat stress, allowing these cultivars to maintain a higher *J*_max_, *V*_cmax_ and overall photosynthetic rate at our hot study site.

Leaf chemistry varied between cultivars and changed over the ripening period, but accumulation was significantly different between cultivars only for Mg. Mean Ca, K and Mg concentrations varied significantly between cultivars (Fig. 5). For all cultivars, Ca was the most concentrated mineral at each time point and the most accumulated mineral over time, as observed previously for individual cultivars (e.g. Merlot; Degu *et al.*, 2019). Ca is immobile in the phloem, which limits translocation to the berries and facilitates accumulation in the leaves as berry hydraulics become phloem-dominated at veraison (Hocking *et al.*, 2016). Mg concentrations increased and K concentrations decreased over the season for all cultivars, contrary to previous findings for K accumulating in response to water stress (Patakas *et al.*, 2002; Degu *et al*, 2019) (Fig. 5). Post-veraison competition between the leaf and berry could have driven the decreases in K, because berry osmotic regulation and demand for K increases near harvest (Monder *et al.*, 2021). K also mediates drought responses by assisting with stomatal regulation (Monder *et al.*, 2021) and, notably, Syrah exhibited the highest K concentrations and gas-exchange rates. Mg and K also compete for plant uptake, and the relatively low soil K/Mg ratio at our site (<0.1) could have contributed to the greater accumulation of Mg. Altogether, our findings show that cultivars growing at the same site and grafted to the same rootstock can vary significantly in nutrient content. The mechanisms driving these differences are poorly understood, and these differences were not explained by climate associations (Supplementary Data Table S2). Finally, TAA and proline content were not significantly different between cultivars or climate groups, contrary to our hypothesis that heat-adapted cultivars would generate osmoprotectant compounds to protect the photochemical machinery from stress.

In sum, contrary to findings for other crops and wild plant species, we did not find that winegrape cultivars have adapted to hotter, drier conditions by increasing osmotic adjustment or reducing osmotic potentials (Bartlett *et al.*, 2012, 2014; Blum *et al.*, 2017). Instead, osmotic potentials were either unrelated or positively correlated with gas exchange, and heat-adapted cultivars exhibited both higher photosynthetic rates and less negative osmotic potentials (Fig. 4; Tables 1 and 2). These findings suggest that differences among cultivars in gas exchange are driven primarily by traits besides the capacity for maintenance of turgor, and that osmotic potentials in grape are more closely related to processes other than leaf water relationships. Increasing photosynthesis in hot conditions emerged as a more promising target for cultivar improvement than reducing osmotic potentials, if breeding programmes build on existing adaptations, but more work is needed to evaluate whether this strategy is beneficial in the new conditions expected from climate change.

## Conclusion

We tested whether leaf osmotic potential and osmotic adjustment, classical water relationship traits that have been highly predictive of drought tolerance in other crops and naturally occurring plant species, have been important drivers of environmental diversification for winegrapes. We hypothesized that grape cultivars have adapted to hotter, drier growing regions by using greater osmotic adjustment and more negative osmotic potentials to improve maintenance of turgor and reduce vulnerability to wilting, hydraulic dysfunction and stomatal closure. Our seven geographically diverse focal cultivars varied significantly in mean osmotic potentials and osmotically adjusted significantly from the onset of ripening (veraison to harvest), but the cultivars associated with the hottest, driest regions exhibited the least negative osmotic potentials, contrary to our hypotheses. Osmotic potentials were either uncorrelated or positively correlated with gas exchange, indicating that grapevines have not improved gas exchange in hot conditions by increasing the capacity for maintenance of turgor. Instead, grapevine osmotic potentials could be related more closely to nutrient storage or sugar translocation. Photosynthesis, but not stomatal conductance, was significantly higher in the heat-adapted cultivars at our hot study site. Future studies should test whether this relationship reflects selection for more heat-tolerant photochemical machinery in the hot-climate cultivars. Leaf chemistry was not related to climate, indicating that heat-adapted cultivars did not maintain greater photosynthesis through increased production of osmoprotectants. Overall, these findings suggest that maintenance of leaf turgor is not a primary limitation on grapevine adaptation to hot, dry atmospheric growing conditions, and that other traits, including photochemical heat tolerance, would be a more promising focus for efforts at cultivar improvement.

## Supplementary Material

mcae032_suppl_Supplementary_Materials

## References

[CIT0001] Alsina MM , de Herralde F, Aranda X, Savé R, Biel C. 2007. Water relations and vulnerability to embolism in eight grapevine cultivars. Vitis 46: 1–6.

[CIT0002] Alston, JM, Sambucci, O. 2019. Grapes in the world economy. In: Cantu D, Walker MA, eds. The grape genome. Cham: Springer International, 1–24.

[CIT0003] Anderson K , Nelgen S. 2020. Which winegrape varieties are grown where? A global empirical picture. Adelaide: University of Adelaide Press.

[CIT0004] Baltzer JL , Davies SJ, Bunyavejchewin S, Noor NSM. 2008. The role of desiccation tolerance in determining tree species distributions along the Malay–Thai Peninsula. Functional Ecology 22: 221–231.

[CIT0005] Bartlett MK , Gabriela S. 2021. Temperature and evaporative demand drive variation in stomatal and hydraulic traits across grape cultivars. Journal of Experimental Botany 72: 1995–2009. 33300576 10.1093/jxb/eraa577

[CIT0006] Bartlett MK , Christine S, Lawren S. 2012. The determinants of leaf turgor loss point and prediction of drought tolerance of species and biomes: a global meta-analysis. Ecology Letters 15: 393–405. 22435987 10.1111/j.1461-0248.2012.01751.x

[CIT0007] Bartlett MK , Zhang Y, Kreidler N, et al. 2014. Global analysis of plasticity in turgor loss point, a key drought tolerance trait. Ecology Letters 17: 1580–1590. 25327976 10.1111/ele.12374

[CIT0008] Bartlett MK , Klein T, Jansen S, Choat B, Sack L. 2016. The correlations and sequence of plant stomatal, hydraulic, and wilting responses to drought. Proceedings of the National Academy of Sciences of the United States of America 113: 13098–13103. 27807136 10.1073/pnas.1604088113 PMC5135344

[CIT0009] Blum A. 2017. Osmotic adjustment is a prime drought stress adaptive engine in support of plant production. Plant, Cell & Environment 40: 4–10. 10.1111/pce.12800 27417527

[CIT0056] Charrier G , Sylvain D, Jean-Christophe D, et al. 2018. Drought Will Not Leave Your Glass Empty: Low Risk of Hydraulic Failure Revealed by Long-Term Drought Observations in World’s Top Wine Regions. *Science Advances* 4: eaao6969. 29404405 10.1126/sciadv.aao6969 PMC5796794

[CIT0011] Chaves M , Harley PC, Tenhunen JD, Lange OL. 1987. Gas exchange studies in two Portuguese grapevine cultivars. Physiologia Plantarum 70: 639–647.

[CIT0012] Cortés AJ , Lópes-Hernández F. 2021. Harnessing crop wild diversity for climate change adaptation. Genes 12: 783. 34065368 10.3390/genes12050783 PMC8161384

[CIT0013] Costa JM , Ortu O MF, Lopes CM, Chavez CM. 2012. Grapevine varieties exhibiting differences in stomatal response to water deficit. Functional Plant Biology 39: 179–189. 32480772 10.1071/FP11156

[CIT0014] DaMatta FM , Grandis A, Arenque BC, Buckeridge MS. 2010. Impacts of climate changes on crop physiology and food quality. Food Research International 43: 1814–1823.

[CIT0015] Dayer S , Herrera JC, Dai Z, et al. 2020. The sequence and thresholds of leaf hydraulic traits underlying grapevine varietal differences in drought tolerance. Journal of Experimental Botany 71: 4333–4344. 32279077 10.1093/jxb/eraa186 PMC7337184

[CIT0016] Degu A , Hochberg U, Wong DCJ, et al. 2019. Swift metabolite changes and leaf shedding are milestones in the acclimation process of grapevine under prolonged water stress. BMC Plant Biology 19: 69. 30744556 10.1186/s12870-019-1652-y PMC6371445

[CIT0017] Fraga H , García de Cortázar Atauri I, Malheiro AC, Santos JA. 2016. Modelling climate change impacts on viticultural yield, phenology and stress conditions in Europe. Global Change Biology 22: 3774–3788. 27254813 10.1111/gcb.13382

[CIT0018] Gagneul D , Aïnouche A, Duhazé D, Lugan R, Larher FR, Bouchereau A. 2007. A reassessment of the function of the so-called compatible solutes in the halophytic Plumbaginaceae *Limonium latifolium*. Plant Physiology 144: 1598–1611. 17468212 10.1104/pp.107.099820 PMC1914112

[CIT0019] Gallo AE , Perez Peña JE, Prieto JA. 2021. Mechanisms underlying photosynthetic acclimation to high temperature are different between *Vitis vinifera* cv. Syrah and Grenache. Functional Plant Biology 48: 342–357. 33278910 10.1071/FP20212

[CIT0021] Greer DH. 2018. The short-term temperature-dependency of CO_2_ photosynthetic responses of two *Vitis vinifera* cultivars grown in a hot climate. Environmental and Experimental Botany 147: 125–137.

[CIT0022] Hasegawa T , Wakatsuki H, Ju H, et al. 2022. A global dataset for the projected impacts of climate change on four major crops. Scientific Data 9: 58. 35173186 10.1038/s41597-022-01150-7 PMC8850443

[CIT0023] Henry C , John GP, Pan R, et al. 2019. A stomatal safety-efficiency trade-off constrains responses to leaf dehydration. Nature Communications 10: 3398. 10.1038/s41467-019-11006-1 PMC6667445 31363097

[CIT0024] Herrera JC , Calderan A, Gambetta GA, et al. 2022. Stomatal responses in grapevine become increasingly more tolerant to low water potentials throughout the growing season. The Plant Journal: for Cell and Molecular Biology 109: 804–815. 34797611 10.1111/tpj.15591

[CIT0025] Hocking B , Tyermna SD, Burton RA, Gilliham M. 2016. Fruit calcium: transport and physiology. Frontiers in Plant Science 7: 569. 27200042 10.3389/fpls.2016.00569 PMC4850500

[CIT0052] Hsiao TC , Acevedo E, Fereres E, Henderson DW. 1976. Water Stress, Growth, and Osmotic Adjustment. Philosophical Transactions of the Royal Society of London. *Series B, Biological Sciences* 273: 479–500.

[CIT0026] Jones GV , White MA, Cooper OR, et al. 2004. *Climate and Wine: Quality Issues in a Warmer World*. https://www.researchgate.net/profile/GregoryJones/publication/267855409_Climate_and_Wine_Quality_Issues_in_a_Warmer_World/links/55eda6e108aef559dc42222c/Climate-and-Wine-Quality-Issues-in-a-Warmer-World.pdf (11 February 2024, date last accessed).

[CIT0028] Jones MM , Turner NC. 1980. Osmotic adjustment in expanding and fully expanded leaves of sunflower in response to water deficits. Australian Journal of Plant Physiology 7: 181–192.

[CIT0029] Lamarque LJ , Delmas CEL, Charrier G, et al. 2023. Quantifying the grapevine xylem embolism resistance spectrum to identify varieties and regions at risk in a future dry climate. Scientific Reports 13: 7724. 37173393 10.1038/s41598-023-34224-6 PMC10181993

[CIT0030] Lobell DB , Field CB, Cahill KN, Bonfils C. 2006. Impacts of future climate change on California perennial crop yields: model projections with climate and crop uncertainties. Agricultural and Forest Meteorology 141: 208–218.

[CIT0032] Martorell S , Medrano H, Tomàs M, Escalona JM, Flexas J, Diaz-Espejo A. 2015. Plasticity of vulnerability to leaf hydraulic dysfunction during acclimation to drought in grapevines: an osmotic-mediated process. Physiologia Plantarum 153: 381–391. 25132228 10.1111/ppl.12253

[CIT0033] Medrano H , Escalona JM, Cifre J, Bota J, Flexas J. 2003. A ten-year study on the physiology of two Spanish grapevine cultivars under field conditions: effects of water availability from leaf photosynthesis to grape yield and quality. Functional Plant Biology 30: 607–619. 32689046 10.1071/FP02110

[CIT0053] Meyer GA , Keliher PN. 1992. An Overview of Analysis by Inductively Coupled Plasma-Atomic Emission Spectrometry. *Inductively Coupled Plasma in Analytical Atomic Spectrometry*, 2nd: 473–516.

[CIT0034] Monder H , Maillard M, Chérel I, et al. 2021. Adjustment of K^+^ fluxes and grapevine defense in the face of climate change. International Journal of Molecular Sciences 22: 10398. 34638737 10.3390/ijms221910398 PMC8508874

[CIT0035] Morgan JM. 1984. Osmoregulation and water stress in higher plants. Annual Review of Plant Physiology 35: 299–319.

[CIT0036] Moutinho-Pereira J Magalhães N , Gonçalves B, Bacelar E, Brito M, Correia C. 2007. Gas exchange and water relations of three *Vitis vinifera* L. cultivars growing under Mediterranean climate. Photosynthetica 45: 202–207.

[CIT0037] Paleari L , Li T, Yang Y, et al. 2022. A trait-based model ensemble approach to design rice plant types for future climate. Global Change Biology 28: 2689–2710. 35043531 10.1111/gcb.16087

[CIT0038] Palliotti A , Tombesi S, Frioni T, et al. 2015. Physiological parameters and protective energy dissipation mechanisms expressed in the leaves of two *Vitis vinifera* L. genotypes under multiple summer stresses. Journal of Plant Physiology 185: 84–92. 26310367 10.1016/j.jplph.2015.07.007

[CIT0039] Patakas A , Noitsakis B. 1999. Osmotic adjustment and partitioning of turgor responses to drought in grapevines leaves. American Journal of Enology and Viticulture 50: 76–80.

[CIT0040] Patakas A , Nikolaou N, Zioziou E, Radoglou K, Noitsakis B. 2002. The role of organic solute and ion accumulation in osmotic adjustment in drought-stressed grapevines. Plant Science 163: 361–367.

[CIT0041] Rodriguez-Dominguez CM , Buckley TN, Egea G, et al. 2016. Most stomatal closure in woody species under moderate drought can be explained by stomatal responses to leaf turgor. Plant, Cell & Environment 39: 2014–2026. 10.1111/pce.12774 27255698

[CIT0054] Sah RN , Miller RO. 1992. Spontaneous Reaction for Acid Dissolution of Biological Tissues in Closed Vessels. *Analytical Chemistry* 64: 230–233. 1319690 10.1021/ac00026a026

[CIT0042] Scoffoni C , Albuquerque C, Cochard H, et al. 2018. The causes of leaf hydraulic vulnerability and its influence on gas exchange in *Arabidopsis thaliana*. Plant Physiology 178: 1584–1601. 30366978 10.1104/pp.18.00743 PMC6288733

[CIT0044] Sorek Y , Greenstein S, Netzer Y, Shtein I, Jansen S, Hochberg U. 2021. An increase in xylem embolism resistance of grapevine leaves during the growing season is coordinated with stomatal regulation, turgor loss point and intervessel pit membranes. The New Phytologist 229: 1955–1969. 33098088 10.1111/nph.17025

[CIT0045] Tyree MT , Ewers FW. 1991. The hydraulic architecture of trees and other woody plants. The New Phytologist 119: 345–360.

[CIT0046] Vivin P , Lebon E, Dai Z, et al. 2017. Combining ecophysiological models and genetic analysis: a promising way to dissect complex adaptive traits in grapevine. OENO One 51: 181–189.

[CIT0055] Wallis CM , Chen, J, Civerolo, EL. 2012. Zebra Chip-Diseased Potato Tubers Are Characterized by Increased Levels of Host Phenolics, Amino Acids, and Defense-Related Proteins. *Physiological and Molecular Plant Pathology* 78: 66–72.

[CIT0048] Williams LE. 2014. Determination of evapotranspiration and crop coefficients for a Chardonnay vineyard located in a cool climate. American Journal of Enology and Viticulture 65: 159–169.

[CIT0049] Yuanyuan M , Yali Z, Jiang L, Hongobo S. 2009. Roles of plant soluble sugars and their responses to plant cold stress. African Journal of Biotechnology 8: 2004–2010.

[CIT0051] Zivcak M , Brestic M, Sytar O. 2016. Osmotic adjustment and plant adaptation to drought stress. In: Hossain MA, Hussain Wani S, Bhattacharjee S, Burritt DJ, Phan Tran LS, eds. *Drought stress tolerance in plants*, Vol 1. *Physiology and biochemistry*. Cham: Springer International, 105–143.

