# Supplementary material for: Grape cultivars adapted to hotter, drier growing regions exhibit greater photosynthesis in hot conditions despite less drought-resistant leaves
Source: Ann Bot. 2024 Mar 13;134(2):205–18. doi: 10.1093/aob/mcae032 (PMC11232511; doi:10.1093/aob/mcae032)
Supplement: mcae032_suppl_Supplementary_Materials [file mcae032_suppl_supplementary_materials.docx]

| **Table S1**: Type = II ANOVA Results. Significance table of measured variables, date, variety, and their interaction (Date x Variety) | | | |
| --- | --- | --- | --- |
| **Variable** | **Date** | **Variety** | **DxV** |
| Ca | * | * | NS |
| K | * | * | NS |
| Na | NS | NS | NS |
| Mg | * | * | * |
| Total Ions | * | NS | NS |
| Proline | NS | NS | NS |
| TAA | * | NS | NS |
| Osmometry | * | * | NS |
| gs | * | * | NS |
| A | * | * | NS |
| PD | NS | NS | NS |
| MD | * | * | NS |

Asterisks * indicate significant results (P<0.05). NS indicates Not Significant

**Table S4**: Type = III ANOVA Results. Significance table of measured variables, date, variety, and their interaction (Date x Climate)

| Variable | Date | Climate | DxC |
| --- | --- | --- | --- |
| Ca | * | NS | NS |
| K | * | NS | NS |
| Na | NS | NS | NS |
| Mg | * | NS | NS |
| Total Ions | * | NS | NS |
| Proline | NS | NS | NS |
| TAA | * | NS | NS |
| Osmometry | * | * | NS |
| gs | * | * | NS |
| A | * | * | NS |
| PD | * | NS | NS |
| MD | * | NS | NS |

Asterisks * indicate significant results (P<0.05). NS indicates Not Significant. There were no differences between type 2 and 3 models.
